# Posttraumatic stress disorder in disaster-exposed youth: examining diagnostic concordance and model fit using ICD-11 and DSM-5 criteria

**DOI:** 10.1186/s12887-024-05317-6

**Published:** 2025-01-11

**Authors:** BreAnne A. Danzi, Ellen A. Knowles, Rachel C. Bock

**Affiliations:** Department of Psychology, University of South Dakota, 414 E. Clark St, Vermillion, SD USA

**Keywords:** Posttraumatic stress disorder, ICD-11, DSM-5, Children and adolescents, Diagnostic criteria, Assessment

## Abstract

**Background:**

Competing definitions of posttraumatic stress disorder (PTSD) have been proposed by ICD-11 and DSM-5; it is unclear which diagnostic model works best for children and adolescents. Although other studies have predicted the impact of these models by approximating the criteria using older measures, this study advances the research by comparing measures designed to assess ICD-11 and DSM-5 criteria in hurricane-exposed youth. This study evaluates ICD-11 and DSM-5 (both the standard and preschool-age) diagnostic models by identifying diagnostic rates, evaluating diagnostic concordance, investigating the predictive value of constructs associated with PTSD (demographics, disaster threat and exposure, functional impairment), and examining model fit.

**Method:**

The sample was exposed to Hurricane Ian (2022), a deadly Category 5 hurricane. Parents reported on disaster exposure and their child’s PTSD symptoms (*n* = 152; ages 7–17) using the International Trauma Questionnaire for Children and Adolescents Caregiver Version (ITQ-CG) for ICD-11 criteria and UCLA PTSD Reaction Index for DSM-5, Parent/Caregiver Report Version (RI-5) for DSM-5 criteria.

**Results:**

ICD-11 PTSD symptom criteria rates were 24% and dropped to 20% when the impairment criterion was added. PTSD symptom criteria rates were 11% (10% with impairment) for DSM-5 and 13% (12% with impairment) for DSM-5 Preschool. ICD-11 rates were higher than DSM-5 and DSM-5 Preschool rates. There was no difference between DSM-5 and DSM-5 Preschool rates of PTSD. There was moderate to substantial concordance between ICD-11 and the DSM-5 models. All diagnostic models were associated with exposure and impairment, but only ICD-11 was associated with threat. ICD-11 was the only one to evidence acceptable model fit.

**Conclusions:**

Using the ITQ-CG and RI-5 to assess PTSD in youth, results showed higher PTSD diagnostic rates for ICD-11 than DSM-5; this contradicts prior findings (based off approximated ICD-11 criteria) and seems largely due to differences in symptom thresholds used by the two measures. The ITQ-CG exhibited excellent model fit and was associated with several constructs important to PTSD.

## Background

Trauma exposure is common in youth, with 60% experiencing a traumatic event [56], and 16% showing symptoms of posttraumatic stress disorder (PTSD) following exposure [3]. The incidence of weather-related traumatic experiences is on the rise, increasing 81% from 1980 to 2022 [19]. Given the large number of youth affected by disasters, accurate screening and assessment of PTSD is essential for identifying psychological needs and triaging treatment resources post-disaster. However, major discrepancies exist for how PTSD is conceptualized and assessed. Competing definitions of PTSD have been proposed in the International Classification of Diseases, 11th edition (ICD-11) by the World Health Organization (WHO) in 2019 and the Diagnostic and Statistical Manual of Mental Disorders, Fifth Edition (DSM-5) by the American Psychiatric Association (APA) in 2013, with a text revision released in 2022. The DSM-5 criteria take a broad approach to PTSD with many symptoms (including symptoms shared with other disorders) across four symptom clusters, whereas the ICD-11 criteria focus on few symptoms considered central to PTSD with three symptom clusters [20]. Although studies have estimated the impact of these changes using approximated forms of the diagnostic criteria (e.g., [57]), there is a dearth of research using validated measures designed to assess the ICD-11 and DSM-5 PTSD criteria in youth. The purpose of this study is to address this gap in the literature by using validated measures to compare diagnostic rates and model fit between the ICD-11 and DSM-5 PTSD definitions. Furthermore, this study focuses on comparing parent-report measures, which are frequently used in research and clinical practice to provide collateral data on the child’s symptoms.

Competing conceptualizations of PTSD have been of high interest in the literature in recent years. Almost all the research in adults has found that DSM-5 yields higher PTSD diagnostic rates than ICD-11 (e.g., [40]). The picture grows more complicated when considering children and adolescents. Most studies of youth have found similar diagnostic rates between ICD-11 and DSM-5, including two U.S. samples of hurricane-exposed preadolescent children [13], terrorism-exposed adolescents in Norway [23], youth in Austrian foster care [7], youth at European emergency departments [18], Chinese adolescents with depression [26], Chinese earthquake-exposed adolescents and a Chinese community sample of adolescents [57]. However, only two of these studies [7, 26] used separate measures that were specifically designed for ICD-11 and DSM-5. One study of youth in mental health clinics found that DSM-5 rates were higher than ICD-11 [46]. This finding is consistent with trends in other research, where DSM-5 rates were higher than ICD-11 but the difference did not reach statistical significance [7]. Further, the study of terrorism-exposed adolescents had differing findings depending on the time elapsed since the trauma and the reporter (self-report versus parent-report): when using parent-report, DSM-5 rates were higher than ICD-11 [23]. However, even studies that identified similar diagnostic rates found that different children were diagnosed with PTSD under the two models, evidenced by low rates of agreement (28—49%) between diagnostic models [13, 46, 57].

The factor structure of these models has also been investigated. Extant research has demonstrated support for the four-factor DSM-5 model in youth (e.g., [17, 30, 39, 52]). Yet, some evidence suggests this model is a poor fit due to unexplained variance between symptoms that are not aptly accounted for within the model [38]. Indeed, alternative DSM-5 models that include additional factors have evidenced superior fit in several cases (e.g., [10, 46]). Researchers have also evaluated the factor structure of ICD-11 PTSD models, though most of such research has investigated models of Complex PTSD in adults (see [43] for review [47]). Fewer studies have focused on the ICD-11 PTSD factor structure in youth,two of such studies found support for the standard three-factor model [30, 46] and one supported an alternative two-factor model [24], though this sample also included young adults. As such, additional research is warranted to determine the appropriate factor structure for PTSD in youth.

In addition to the standard DSM-5 criteria for adults and older youth, APA released separate PTSD criteria for children ages six and younger (i.e., the “DSM-5 Preschool” criteria).

These criteria were designed to be a superior fit for young children, based on research identifying unique PTSD symptom profiles among young children exposed to trauma [16]. Specifically, the DSM-5 Preschool criteria place less emphasis on internalized, cognitively-sophisticated symptoms that may be too advanced for children and instead focus on externalized, behaviorally-focused symptoms. Although originally intended for young children, it may be the case that these modifications to the diagnostic criteria are advantageous for older children too. In fact, emerging evidence suggests that the DSM-5 Preschool model may better capture the trauma responses of youth (older than age six) compared to the other diagnostic criteria designed for adults. Specifically, the DSM-5 Preschool criteria yielded higher diagnostic rates in a hurricane-exposed sample [14], a terrorism-exposed sample [15], and a treatment-seeking sample of varying trauma types [37]. The DSM-5 Preschool criteria has also demonstrated comparable model fit as the standard DSM-5 criteria [14]. While this model was originally designed to capture the unique symptom profiles of young children, these studies suggest that the model warrants continued investigation for use with older youth as well. Utilizing this model with youth older than six and comparing results to other models, such as the DSM-5 standard criteria and ICD-11, can help to provide further information about whether the DSM-5 Preschool model is only suitable for young children or if it equally captures PTSD symptoms in youth across development.

The current literature is limited by most studies using a post-hoc methodology in which measures designed to assess DSM-5 criteria were rearranged to resemble ICD-11 criteria [13, 15, 18, 23, 24, 30, 46, 57]. While this approach was appropriate at the time, formal measures that are specifically designed for the ICD-11 have since been developed. Thus, the next step to advance this research is to compare these diagnostic models using model-specific, validated measures in youth of varying ages exposed to diverse trauma types [8].

This study addresses this research gap by utilizing the International Trauma Questionnaire for Children and Adolescents Caregiver Version (ITQ-CG; [35]), which was designed to measure ICD-11 criteria in youth. The self-report version of this measure (ITQ-CA) has been used in only two studies comparing diagnostic models: one study used the German version in foster care youth [7] and the other used the Chinese version in a sample of treatment-seeking adolescents with depression [26]. Other research has investigated the factor structure of the ITQ-CA in youth exposed to abuse or community samples of mixed trauma types (e.g., [25, 28, 34, 44]). This study is the first to date to evaluate the caregiver-report ITQ-CG (English version) in a disaster-exposed sample. Gold standard assessment of psychological symptoms in youth incorporates both caregiver- and child-report. Caregiver-reported symptoms are distinct from self-reported symptoms in children, but both are important constructs; thus, it is important to understand the utility of both. Caregiver report may be particularly important when samples include younger children with limited insight or who struggle to articulate their post-trauma symptoms. It is important to note that questionnaire measures are not capable of formally diagnosing PTSD, but are frequently used to identify youth at heightened risk for PTSD or “probable” PTSD. This study compares the performance of caregiver-report questionnaire measures and evaluates both PTSD symptom criteria as well as the PTSD symptom criteria combined with the impairment criterion. This study advances the literature by investigating caregiver-reported PTSD symptoms in children and adolescents and compares this construct between two diagnostic models.

In addition to evaluating how well the measures perform at identifying children with PTSD, the association between the measures and a range of variables found to be linked with the PTSD construct was also examined. Variables often linked with PTSD include demographics (gender, race/ethnicity, age), disaster threat (perceived life threat, fear/distress), disaster exposure (actual life threat), and post-disaster functioning (functional impairment). PTSD has been found to be associated with being female, non-White/non-Hispanic, or younger age in meta-analyses [53, 54]. Threat and exposure are conditions prerequisite to developing PTSD and must be present for a diagnosis of PTSD [1, 58]. In addition to actual threat, perceived life threat (i.e., belief that one’s life is in danger) is considered critical to the development of PTSD [12, 36]. Functional impairment also is regarded as crucial to the PTSD diagnosis [1, 27, 58], and so serves as a useful comparison point for the different models of PTSD symptom criteria in order to evaluate which PTSD model is best at identifying highly impaired children. PTSD symptom criteria with stronger associations to these well-established variables may be more closely linked to the underlying PTSD construct.

The purpose of the current study is to compare DSM-5 and ICD-11 symptom criteria in disaster-exposed youth using validated, model-specific, parent-report measures: the commonly used UCLA PTSD Reaction Index for DSM-5 (RI-5; [41]) and the ITQ-CG. This study also explores the potential impact of using the DSM-5 Preschool criteria with older youth, though no known model-specific measures have been validated in older youth. The study aims were to: 1) compare symptom cluster and diagnostic rates according to ICD-11, DSM-5, and DSM-5 Preschool criteria; 2) examine diagnostic concordance between models; 3) evaluate the predictive value of variables commonly associated with PTSD (demographics, threat, trauma exposure, functional impairment) for each model; and 4) evaluate model fit. In addition, because the ITQ-CG and RI-5 use different thresholds to indicate symptom presence, we conducted post-hoc exploratory analyses to determine if findings changed based on adjusting the symptom thresholds to be analogous.

## Methods

### Participants

Participants were exposed to Hurricane Ian, a Category 5 hurricane that struck Florida, USA in September 2022. Hurricane Ian was the deadliest storm to strike Florida in almost a century and the third costliest cyclone on record, with at least 156 fatalities and $113 billion in damages [6]. Data were collected two to seven months post-disaster. Parents living in the most impacted Florida counties (Lee, Charlotte, Collier, Desoto, Hardee, Highlands, or Sarasota) reported their child’s disaster-related symptoms and functioning.

Parents (*N* = 152) were 77% female and 76% non-Hispanic White, 13% Hispanic/Latine, 9% Black, 1% Asian, and 1% multiracial. The children were ages 7–17 (*M* = 10.82; *SD* = 3.00) and in first through twelfth grade (59% elementary, 21% middle, 20% high school). They were 48% female and 70% non-Hispanic White, 16% Hispanic/Latine, 9% Black, 1% Asian, and 4% multiracial. The sample was largely representative of the racial/ethnic make-up of the included Florida counties [55]. Most parents were ages 25–44 (81%), had a Bachelor’s degree or some college education (70%), and were married (74%). Families’ gross annual income was less than $50 K for 32%, $50 K-$100 K for 49%, and over $100 K for 19%. During Hurricane Ian, 56% resided in a mandatory evacuation zone.

### Procedures

This study was approved by the University of South Dakota Institutional Review Board. Participants provided informed consent prior to participating in the study. Eligibility criteria included residence in one of seven Florida counties most impacted by Hurricane Ian and being the parent/primary caregiver of at least one child under 18 years of age. Recruitment methods included posting flyers in family-orientated facilities, social media posts, and listservs to schools, churches, daycares, and other community-based organizations. Participants completed a 15–20-minute online assessment battery. To verify eligibility and prevent inattention or fraudulent responses, several checks were utilized based on current recommendations [2]: 1) zip code consistent with reported county; 2) child’s date of birth consistent with reported age; 3) provided a school name located in/near county; 4) selecting “911” as their emergency telephone number; and 5) favorite color attention check item (see [2] for more detail). Participants who passed four out of five of these checks and provided a valid mailing address located within an eligible county (one response per household was permitted) received a $5.00 gift card and a workbook on helping children cope after hurricanes.

### Measures

#### Demographics

Gender, race, ethnicity, age, and education level were obtained for the parents and the children, as well as the family’s gross annual income, marital status, and home address at the time of the hurricane.

#### International Trauma Questionnaire

PTSD as defined by ICD-11 was evaluated using the International Trauma Questionnaire Child and Adolescent Caregiver Report Version (ITQ-CG; [9, 35]), which is appropriate for youth ages 7–17. The child and adolescent version was adapted from the original adult version of the ITQ to increase the comprehensibility of the measure for children and adolescents [9], and was later modified to include caregiver-report [35]. However, the adapted measure maintains much of the same structure and scoring schemes as the original adult measure, which has been widely used and validated among trauma-exposed samples [43]. The child and adolescent version has been used among youth exposed to a range of traumatic events and has proved to be both valid and reliable across contexts (e.g., [7, 25, 28]). Previous studies have emphasized a need to investigate the utility of the caregiver report version, an area which has thus far been neglected in the literature [25]. The ITQ-CG PTSD symptoms are assessed using six items that ask caregivers to report on PTSD symptoms using a 5-point Likert scale (0 = *Never,* 1 = *A little bit,* 2 = *Sometimes,* 3 = *A lot,* 4 = *Almost Always*) during the past month (total scores range 0–24). The diagnostic algorithm uses a score of 2 + (“*Sometimes*”) to indicate symptom presence and requires at least one symptom to be present in each of the three symptom clusters (Re-experiencing, Avoidance, Arousal). The ITQ-CG also includes a functional impairment subscale of five items that evaluate whether PTSD symptoms interfered with the child’s ability to function with friends, family, school, hobbies, or with their general happiness. Internal consistency was α = 0.915.

#### UCLA PTSD Reaction Index for DSM-5

PTSD as defined by DSM-5 was evaluated using the UCLA PTSD Reaction Index for DSM-5, Parent/Caregiver Version (RI-5; [41]), which is among the most commonly used and well-validated DSM-5 PTSD measures for youth ages 7–17 and has demonstrated good internal consistency and validity among diverse samples internationally [17]. The RI-5 has been used in previous research to assess DSM-5 PTSD symptoms in samples exposed to natural disasters and other traumatic events (e.g., [11, 32, 51]). Its capacity to maintain validity and reliability across cultures and trauma types demonstrates strong evidence for its effectiveness in identifying PTSD symptomatology in trauma-exposed youth. Furthermore, the Parent/Caregiver Version of the RI-5 has demonstrated sound reliability with the Self-Report Version, thus providing evidence for its utility in assessing youth PTSD symptoms through caregiver report [42]. Parents reported on the frequency of their child’s PTSD symptoms during the past month using a 5-point Likert scale (0 = *None*, 1 = *Little*, 2 = *Some,* 3 = *Much*, 4 = *Most*). A threshold of 3 + is primarily used to indicate symptom presence, with the exception of three items that use a threshold of 2 + . The RI-5 also includes functional impairment items that evaluate if the symptoms caused problems at home, in school, with relationships, or other aspects of development. Standard scoring instructions were utilized to determine if criteria for each of the four DSM-5 clusters (Re-experiencing, Avoidance, Cognitions/Mood, Arousal) and PTSD diagnosis was met. Internal consistency was α = 0.971.

Consistent with most prior research in this area (e.g., [14]), the RI-5 was also used to evaluate the DSM-5 Preschool criteria by adapting the RI-5 scoring to a three-factor model of Re-experiencing, Arousal, and a combined cluster of Avoidance and alterations in Cognitions/Mood. One or more symptoms are required to meet the Re-experiencing cluster, two or more symptoms are needed for the Arousal cluster, and one or more symptoms are needed for the combined Avoidance and Cognitions/Mood cluster.

#### Hurricane-Related Traumatic Experiences

Disaster threat and exposure were evaluated using the Hurricane-Related Traumatic Experiences (HURTE-II; [31]). Perceived life threat was assessed by a binary item asking if the child thought they might die from the hurricane (1 = *Yes*, 0 = *No*). Hurricane-related fear/distress was a summed score of three items on a 4-point Likert scale (0 = *Not at All* to 3 = *A Whole Lot*). Internal consistency for this subscale was α = 0.679. Actual life threat was assessed by summing eight items (1 = *Yes*, 0 = *No*) about life-threatening experiences during the hurricane (e.g., child injured, child hit by flying debris, flood exposure). Internal consistency for this subscale was α = 0.776.

### Statistical analyses

Statistical analyses were conducted using IBM SPSS Statistics v28 and R v4.1 using the lavaan package [45]. Missing data was < 1% and handled with mean imputation. For Aim 1, PTSD symptom criteria and symptom cluster rates were compared for statistically significant differences across models using exact McNemar’s tests. For Aim 2, diagnostic concordance was evaluated using Cohen’s Kappa and interpreted based on recommendations [33]. For Aim 3, hierarchical logistic regression analyses, which allow for the ability to see the added effect of a predictor variable while controlling for other variables, were used to evaluate the relationship between variables well-established to be associated with the PTSD construct and each of the diagnostic models (met PTSD symptom criteria vs. did not meet PTSD symptom criteria). Demographics (gender, race/ethnicity, age) were entered first, followed by disaster threat (perceived life threat, fear/distress), then disaster exposure (actual life threat), concluding with post-disaster functioning (functional impairment). The disaster threat and exposure items were from the HURTE-II. To allow the PTSD symptom criteria to be compared against a consistent measure of impairment, the ITQ-CG functional impairment subscale was used for the logistic regression. Confirmatory factor analyses were conducted to determine model fit. Three models were tested: a three-factor (Re-experiencing; Arousal; Avoidance) ICD-11 PTSD model; a four-factor (Re-experiencing; Avoidance; Cognitions/Mood; Arousal) DSM-5 PTSD model, and a three-factor (Re-experiencing; Arousal; Avoidance/Mood) DSM-5 Preschool model. Models were specified using weighted least squares mean–variance adjusted (WLSMV) estimation given the ordinal nature of the data. Model fit was evaluated using the following guidelines [29]: a nonsignificant chi squared (χ^2^ ) value; Comparative Fit Index (CFI)[4] ≥ 0.90; Root Mean Square Error of Approximation (RMSEA) [50] ≤ 0.06; and Standardized Root Mean Square Residual (SRMR) ≤ 0.08.

## Results

Descriptives and internal consistencies for the symptom clusters and total PTSD scores, according to each diagnostic model, are displayed in Table 1. For DSM-5, the PTSD mean for the sample fell below the clinical cutoff (RI-5 score of 35). For ICD-11, we are unaware of a proposed cutoff score for the ITQ-CG, but the sample mean fell within the lower quartile of the possible range for PTSD. For all three models, the internal consistencies for the total scale fell within the excellent range (Cronbach’s α > 0.90). Internal consistencies fell within the good to excellent range for all the symptom clusters except the DSM-5 Avoidance cluster, which fell within the acceptable range.

**Table 1:** Means, standard deviations, and cronbach’s alphas for PTSD symptom clusters and total scores

	ICD-11 (ITQ-CG)		DSM-5 (RI-5)		DSM-5 Preschool (RI-5)	
	*M* (*SD*)	α	*M* (*SD*)	α	*M* (*SD*)	α
Re-experiencing	1.49 (1.92)	.835	4.39 (4.85)	.913	4.39 (4.85)	.913
Avoidance	1.83 (2.15)	.800	1.95 (2.15)	.747	3.92 (4.65)	.895
Cognitions/Mood	–	–	6.64 (6.78)	.950		
Arousal	1.78 (2.08)	.817	6.13 (5.37)	.877	5.37 (4.62)	.851
PTSD total scale	5.10 (5.62)	.915	19.12 (17.84)	.971	13.68 (13.13)	.951

### PTSD rates

ICD-11 symptom criteria for PTSD were met by 24% of the sample (*n* = 36). When the impairment criterion was added, this rate dropped by six cases, yielding a rate of 20% (*n* = 30). DSM-5 symptom criteria were met by 11% of the sample (*n* = 16); this rate dropped by one case when the impairment criterion was added, yielding a rate of 10% (*n* = 15). The DSM-5 Preschool symptom criteria were met by 13% of the sample (*n* = 20) and this dropped by two cases when the impairment criterion was added, yielding a rate of 12% (*n* = 18).

The rates were compared using exact McNemar’s tests. The ICD-11 symptom criteria rate was higher than the DSM-5 (χ^2^ [1] = 18.05, *p* < 0.001) and DSM-5 Preschool rates (χ^2^ [1] = 11.25, *p* < 0.001). This was also true once the impairment criterion was added (χ^2^ [1] = 13.07, *p* < 0.001; χ^2^ [1] = 8.64, *p* = 0.003) There was no difference between the DSM-5 and DSM-5 Preschool symptom criteria rates (χ^2^ [1] = 1.13, *p* = 0.289); this was also the case when impairment was added (χ^2^ [1] = 0.80, *p* = 0.371). See Table 2.

**Table 2:** Case rates for symptom clusters and PTSD symptom criteria

	ICD-11 *n* (%)	DSM-5 *n* (%)	DSM-5 Preschool *n* (%)
Re-experiencing	49 (32%)	58 (38%)	58 (38%)
Avoidance	52 (34%)^a^	34 (22%)^a^	38 (25%)
Cognitions/Mood	–	37 (24%)	
Arousal	53 (35%)^b^	43 (28%)^c^	35 (23%)^b,c^
PTSD symptom criteria	36 (24%)^a,b^	16 (11%)^a^	20 (13%)^b^
PTSD symptom criteria + impairment	30 (20%)^a,b^	15 (10%)^a^	18 (12%)^b^

^a^significant difference between ICD-11 and DSM-5

^b^significant difference between ICD-11 and DSM-5 Preschool

^c^significant difference between DSM-5 and DSM-5 Preschool

### Symptom cluster rates

For the Re-experiencing cluster, there was no difference in rates between ICD-11, DSM-5, or DSM-5 Preschool. For the Avoidance cluster, more children were identified by ICD-11 compared to DSM-5 (χ^2^ [1] = 10.32, *p* = 0.001). For the Arousal cluster, there was no difference between ICD-11 and DSM-5 (χ^2^ [1] = 2.25, *p* = 0.134), but ICD-11 and DSM-5 each identified more cases than DSM-5 Preschool (χ^2^ [1] = 7.61, *p* = 0.005; χ^2^ [1] = 6.13, *p* = 0.013, respectively). See Table 2 for more detail.

### Diagnostic concordance

There was moderate agreement between ICD-11 and DSM-5 symptom criteria, Cohen’s κ = 0.550, *p* < 0.001. ICD-11 identified all 16 youth identified by DSM-5, plus 20 additional children (44% concordance). When looking at symptom criteria with impairment, there was substantial agreement between ICD-11 and DSM-5: Cohen’s κ = 0.616, *p* < 0.001. There were 15 cases identified by both models and an additional 15 cases identified only by ICD-11 (50% concordance).

There was moderate agreement between ICD-11 and DSM-5 Preschool symptom criteria, Cohen’s κ = 0.570, *p* < 0.001. There were 18 cases identified by both models (47% concordance), 18 cases identified only by ICD-11, and 2 cases identified only by DSM-5 Preschool. When adding impairment to the symptom criteria, there was substantial agreement between ICD-11 and DSM-5 Preschool: Cohen’s κ = 0.658, *p* < 0.001. There were 17 cases identified by both models (55% concordance), 13 cases identified only by ICD-11, and 1 case identified only by DSM-5 Preschool.

Symptom criteria for the DSM-5 models showed substantial agreement, Cohen’s κ = 0.748, *p* < 0.001. There were 14 cases identified by both (64% concordance), 2 cases identified only by DSM-5, and 6 cases identified only by DSM-5 Preschool. When impairment was added, there was almost perfect agreement between the DSM-5 models: Cohen’s κ = 0.830, *p* < 0.001. There were 14 cases identified by both (74% concordance), 1 case identified only by DSM-5, and 4 cases identified only by DSM-5 Preschool.

### Constructs correlated to PTSD

Logistic regression results comparing variables typically associated with PTSD to ICD-11, DSM-5, and DSM-5 Preschool symptom criteria case status (met symptom criteria versus did not meet symptom criteria) are displayed in Table 3. Model summary statistics for Step 1 (demographics), Step 2 (disaster threat), Step 3 (disaster exposure), and Step 4 (post-disaster functioning) are also displayed in Table 3.

**Table 3:** Associations between variables relevant to PTSD and each models’ symptom criteria

	ICD-11		DSM-5		DSM-5 Preschool	
	OR (95% CI)	*p*	OR (95% CI)	*p*	OR (95% CI)	*p*
**Demographics**						
Gender	2.76 (1.22–6.29)	.015	2.12 (.70–6.45)	.187	1.77 (.66–4.73)	.255
Race/ethnicity	1.17 (.50–2.72)	.714	.53 (.14–1.98)	.345	.79 (.27–2.35)	.673
Age	.99 (.86–1.12)	.825	1.00 (.84–1.19)	.979	.99 (.84–1.16)	.859
**Disaster Threat**						
Perceived threat	4.60 (1.89–11.20)	< .001	1.95 (.62–6.19)	.256	2.01 (.71–5.73)	.191
Fear/distress	1.21 (.97–1.52)	.088	1.07 (.81–1.41)	.645	1.24 (.96–1.60)	.103
**Disaster Exposure**						
Actual life threat	1.91 (1.47–2.49)	< .001	1.83 (1.31–2.54)	< .001	1.71 (1.29–2.26)	< .001
**Post-Disaster Functioning**						
Impairment	1.73 (1.27–2.35)	< .001	1.89 (1.27–2.83)	.002	2.37 (1.51–3.72)	< .001
**Model Summary Statistics**						
Demographics	*χ*^2^(3) = 6.60, *p* = .086, Nagelkerke *R*^2^ = .065		*χ*^2^(3) = 2.82, *p* = .038, Nagelkerke *R*^2^ = .038		*χ*^2^(3) = 1.57, *p* = .667, Nagelkerke *R*^2^ = .019	
Disaster Threat	*χ*^2^(5) = 28.42, *p* < .001, Nagelkerke *R*^2^ = .262		*χ*^2^(5) = 5.02, *p* = .414, Nagelkerke *R*^2^ = .067		*χ*^2^(5) = 8.60, *p* = .126, Nagelkerke *R*^2^ = .103	
Disaster Exposure	*χ*^2^(6) = 58.64, *p* < .001, Nagelkerke *R*^2^ = .490		*χ*^2^(6) = 21.19, *p* = .002, Nagelkerke *R*^2^ = .268		*χ*^2^(6) = 25.48, *p* < .001, Nagelkerke *R*^2^ = .288	
Post-Disaster Functioning	*χ*^2^(7) = 71.86, *p* < .001, Nagelkerke *R*^2^ = .576		*χ*^2^(7) = 33.25, *p* < .001, Nagelkerke *R*^2^ = .405		*χ*^2^(7) = 47.26, *p* < .001, Nagelkerke *R*^2^ = .498	

For gender, 0 = girls. For race/ethnicity, 0 = Non-Hispanic White

For ICD-11, four variables significantly predicted PTSD case status and a trend emerged for a fifth variable. For DSM-5 and DSM-5 Preschool, only two variables were associated with PTSD case status. Actual life threat and impairment predicted PTSD case status across all the diagnostic models. However, perceived life threat (and a trend for disaster-related fear/distress) were only associated with PTSD as defined by ICD-11. Demographic variables were not associated with PTSD across the diagnostic models, except for a gender difference in PTSD rates for ICD-11.

### Model fit

**Fig. 1:**
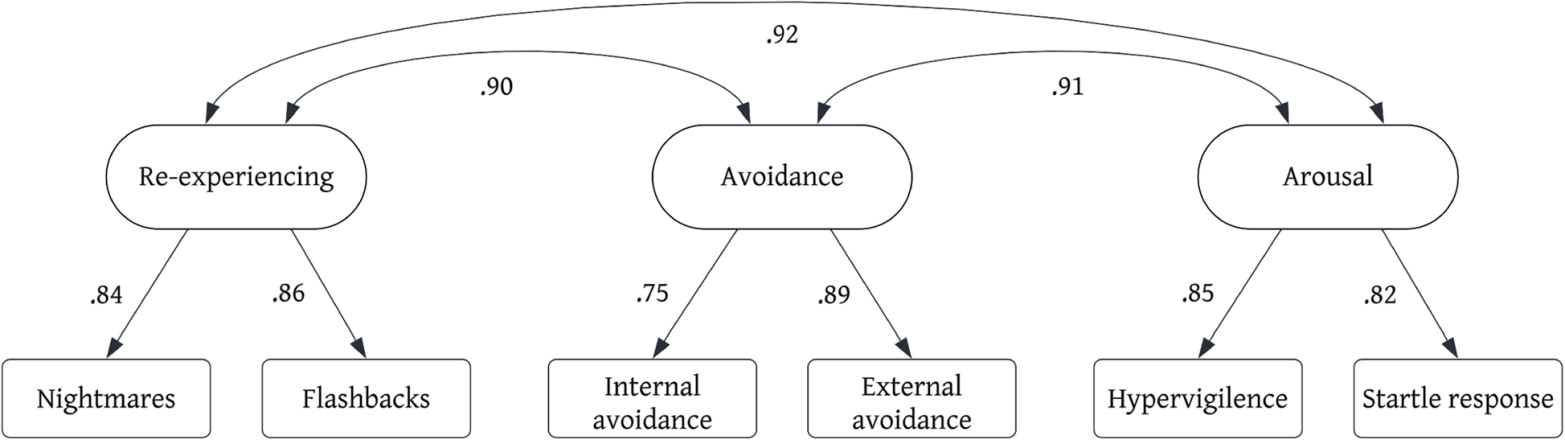
ICD-11

**Fig. 2:**
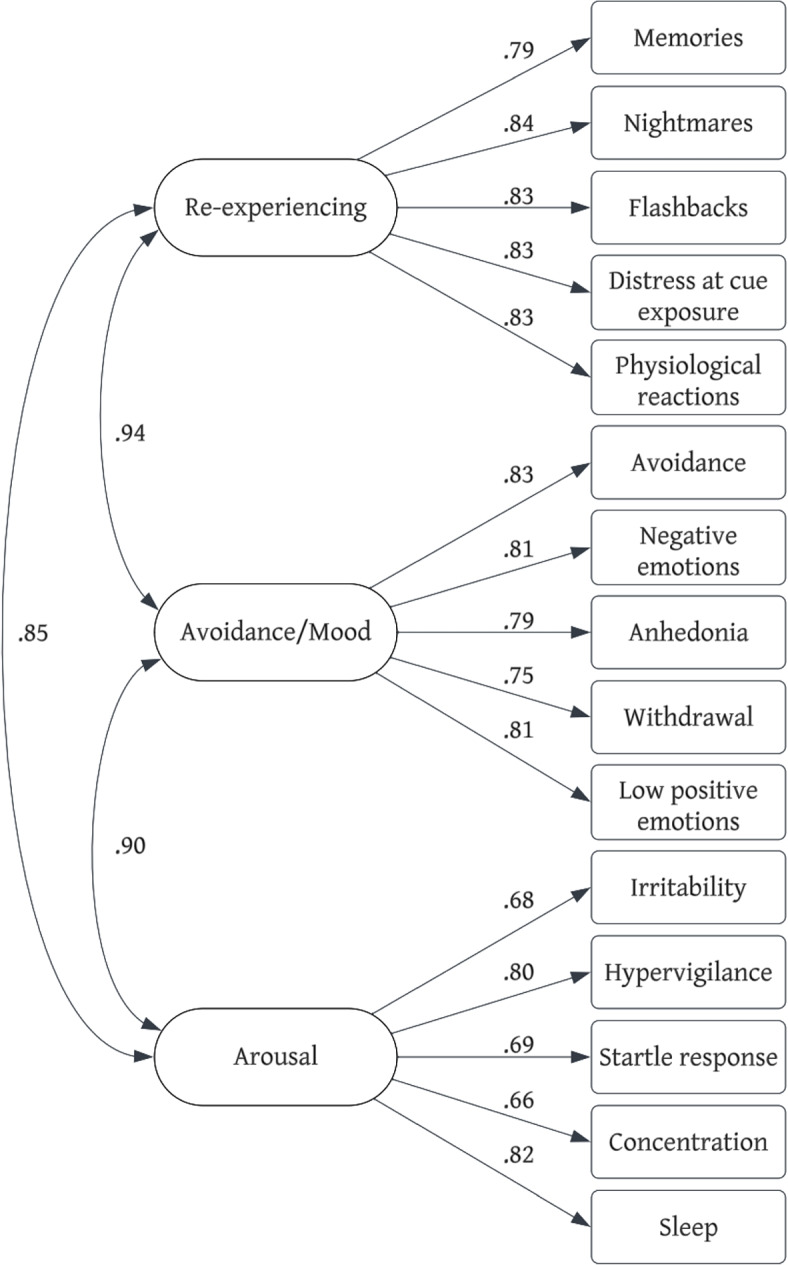
DSM-5 Preschool

Confirmatory factor analyses were conducted with the three-factor ICD-11 PTSD model, four-factor DSM-5 model, and three-factor DSM-5 Preschool model, in accordance with the diagnostic criteria for each model. The three-factor ICD-11 PTSD model was an excellent fit to the data (*χ*^2^(6) = 7.72, *p* = 0.259; CFI = 0.99; RMSEA (95% CI) = 0.04 (0.00, 0.12); SRMR = 0.02). All items loaded onto their respective factors were significant (see Fig. 1). Standardized factor loadings ranged from 0.75 to 0.89 across clusters. The four-factor DSM-5 model could not be identified due to a latent variable covariance matrix error. The Re-experiencing and Avoidance factors were correlated above 1, a result also observed in extant research [38]. As such, we were unable to reliably examine model fit indices for the four-factor DSM-5 model. The three-factor DSM-5 Preschool model chi-squared test was significant (*χ*^2^(87) = 129.12, *p* = 0.002), though approximate-fit indices were each within acceptable bounds (CFI = 0.95; RMSEA (95% CI) = 0.06 (0.03, 0.08); SRMR = 0.06). All item loadings were significant (see Fig. 2). Standardized factor loadings ranged from 0.66 to 0.84 across clusters.

### Exploratory analyses: adjusting symptom thresholds

On an exploratory basis, the threshold for symptom endorsement for the ITQ-CG was raised to resemble the scoring of the RI-5. The ITQ-CG and RI-5 both use a 5-point Likert scale (0–4), but the RI-5 mostly uses a score of 3 + to indicate symptom presence (with a few exceptions), whereas the ITQ-CG uses a score of 2 + to determine symptom presence. After rescoring the ITQ-CG using the higher threshold of 3 + for symptom presence, the PTSD symptom criteria rate dropped from 36 to 11 cases, which was not different from the DSM-5 rate (χ^2^ [1] = 1.23, *p* = 0.267). For the 11 cases, all but one met the impairment criterion. The higher threshold also led to the ICD-11 avoidance cluster resembling the DSM-5 avoidance cluster (31 vs. 34 cases, χ^2^ [1] = 0.17*, p* = 0.677), whereas ICD-11 rates were lower than DSM-5 for the Re-experiencing (19 vs. 58, χ^2^ [1] = 35.22*, p* < 0.001) and Arousal (24 vs. 43, χ^2^ [1] = 9.82*, p* = 0.002) clusters. The altered symptom threshold also changed the ICD-11 rate from being higher than the DSM-5 Preschool rate (previously 36 vs. 20) to being lower than the DSM-5 Preschool rate (11 vs. 20, χ^2^ [1] = 4.27, *p* = 0.039).

## Discussion

An empirically-supported, clinically-useful method of identifying PTSD in children is crucial, especially in the context of large-scale traumas, such as natural disasters. The optimal conceptualization of PTSD has been a source of great debate in the field, heightened by the competing definitions posited by DSM-5 and ICD-11. This study advances this timely area of research by using parent/caregiver report of children’s post-disaster functioning to compare DSM-5 models to ICD-11 using the newly developed ICD-11 measure, the ITQ-CG.

In this study, ICD-11 (as measured by the ITQ-CG) identified twice as many children with PTSD compared to DSM-5 (as measured by the RI-5). This is a marked deviation from the literature, which has found DSM-5 rates to be similar or higher than ICD-11 rates [7, 13, 18, 23, 26, 46, 57]. Additionally, in contrast to prior research [7, 46], no differences emerged in the Re-experiencing cluster rates; only the Avoidance cluster emerged as significantly different. Given that the Avoidance cluster is conceptually similar between ICD-11 and DSM-5, these findings seem largely driven by scoring differences between the two measures being compared. Specifically, the ITQ-CG uses a threshold of “sometimes” (score of 2 on 0–4 scale) to indicate symptom presence whereas the RI-5 primarily uses a threshold of “much” of the time (score of 3 on 0–4 scale). Indeed, after adjusting the ITQ-CG symptom threshold to mirror the RI-5, there was no longer a discrepancy between the Avoidance clusters, which is in line with prior findings. However, it is unclear which symptom threshold is superior. Avoidance is difficult to detect in children [48], so using a lower symptom threshold may resolve this problem. A lower symptom threshold may also be advantageous when using parent/caregiver report because parents/caregivers may observe symptoms less than they actually occur. However, it should be noted that in studies of youth using a single-measure methodology where the same items (with the same scoring thresholds) were used to evaluate DSM-5 vs. ICD-11 diagnostic algorithms, ICD-11 yielded lower or similar prevalence rates compared to DSM-5 algorithms [13, 18, 23, 46, 57]. Thus, it is important to clarify that these findings do not suggest that the ITQ-CG symptom thresholds are too low, but simply that they are lower than the measure being compared in this study, the RI-5. Determining the optimal symptom thresholds to indicate symptom presence is an important direction for future research.

The concordance for PTSD symptom criteria between the ITQ-CG and RI-5 (κ = 0.550; 44% agreement) was similar to what has been previously reported for ICD-11 and DSM-5 (e.g., kappas of 0.22–0.74, 28–49% agreement; [7, 13, 23, 46, 57]). Diagnostic concordance did improve when the functional impairment criterion was added (κ = 0.616,50% agreement), which is positive. However, this remains an area of concern: in addition to the potential for confusion among clinicians, research findings on PTSD etiology and treatment may be systematically skewed between geographical regions predisposed to use DSM-5 vs. ICD-11 measures.

This study also investigated associations between each diagnostic model and theoretically important constructs consistently linked to PTSD [53, 54]. Across models, there were no differences between demographic groups, except for ICD-11 identifying more boys, contrary to expectations [53, 54]. While unexpected, some prior studies have found no differences between boys and girls exposed to trauma [5, 22], further research on this relationship is warranted. Actual life threat, an indicator of trauma exposure, was predictive of PTSD across models and evidenced similar odds ratios (1.71–1.91). Similarly, functional impairment was associated with PTSD symptom criteria across models. Disaster-related threat is where findings diverged. Perceived life threat is theoretically important to PTSD and may be a better predictor of PTSD than actual life threat in youth [36]. Perceived life threat was not predictive of PTSD among the DSM-5 models, although it was predictive of ICD-11 PTSD: children with perceived life threat were over four times more likely to be identified with ICD-11 PTSD. This may be due to the ICD-11 criteria’s focus on symptoms central to PTSD, whereas the DSM-5 criteria include symptoms that overlap with other disorders [20]. Perceived life threat may be most closely tied to those core PTSD symptoms that are emphasized in the ICD-11 criteria. Similarly, there was a trend for children’s hurricane-related fear and distress predicting ICD-11 PTSD.

In addition to yielding associations with perceived threat, the ICD-11 criteria for PTSD exhibited excellent model fit. Conversely, the DSM-5 CFA analysis produced a latent variable covariance matrix error, indicating that underlying measurement assumptions were not met. This outcome was also observed in extant research utilizing the RI-5 in a sample of polyvictimized justice-involved adolescents [38] and similar issues have been noted in the adult literature as well [49]. The heterogeneity of the DSM-5 four-factor structure has been criticized in other research [21] and may be overly complex. In fact, better performance was observed with the DSM-5 Preschool model, which uses many of the same symptoms but applies a three-factor structure. These findings suggest that the more parsimonious models may be advantageous in this sample of hurricane-exposed youth.

Prior research has suggested that the DSM-5 Preschool model may perform well for older youth [14, 15, 37], which was evaluated in this study. The DSM-5 Preschool symptom criteria identified an intermediate number of children between ICD-11 and DSM-5, had moderate concordance with ICD-11, and substantial concordance with DSM-5. When impairment was added, DSM-5 Preschool had substantial agreement with ICD-11 and almost perfect agreement with DSM-5. DSM-5 Preschool performed similarly to DSM-5 in associations with theoretically relevant predictors of PTSD. In terms of model fit, the DSM-5 Preschool criteria did not exhibit the latent variable covariance observed with DSM-5 and demonstrated good fit on three out of four indices. These results provide modest evidence supporting the utility of the DSM-5 Preschool model in older youth. These results are consistent with other research that has found the DSM-5 Preschool criteria to be either more sensitive or similar to standard DSM-5 criteria in older children and adolescents [14, 15, 37]. Further research evaluating how findings may vary as a function of age is warranted.

Several study limitations should be noted. A strength of this study is using recently developed model-specific measures to assess ICD-11 and DSM-5 criteria, which led to different findings than studies using single-measure diagnostic algorithm methodology. However, we were unable to use a model-specific measure for the DSM-5 Preschool criteria, as we are unaware of a well-validated measure of these criteria designed for older youth. As our findings demonstrate, approximations of diagnostic criteria may be less accurate than using model-specific measures. Secondly, although the scope of this study was focused on parent/caregiver report measures, a more complete picture would be provided by obtaining child self-report data as well, as other research has shown differences in how diagnostic models compare using parent- versus self-report [23]. Parents/caregivers may overreport or underreport symptoms in their children, may be influenced in their reports by their own trauma reactions, and may have difficulty observing some symptoms of PTSD (e.g., avoidance). Future research should build on the current study by implementing a multimodal approach of parent-report, self-report, and clinician interview. Third, while the use of online data collection was necessary to facilitate timely data collection in the aftermath of the storm, this may have limited the sample to individuals with internet access. Fourth, the measures were not counterbalanced, with the RI-5 appearing earlier in the survey. Due to this, there is risk of order effects influencing findings. Fifth, this study did not include an evaluation of CPTSD, which limits comparisons to factor analysis studies that used a two-factor second-order model of the ITQ. Future research should evaluate whether the second-order model has superior fit in a hurricane-exposed sample. Finally, although this is the first study to date to investigate the ITQ-CG in the U.S., the sample was majority White and findings may differ across racial/ethnic groups. Additional investigations with more diverse groups is desirable.

Importantly, this study highlights that both the diagnostic criteria used and the symptom threshold used may impact which children are identified with PTSD. It is worth considering whether the ITQ-CG strategy of using fewer, lower-threshold symptoms is preferable to the RI-5 strategy of requiring more, higher-threshold symptoms. From a clinical utility perspective, fewer items reduce assessment burden, which is important when screening for PTSD in the aftermath of a large-scale disaster, and it was telling that ICD-11 managed to identify all the children identified by DSM-5 using this approach, plus additional children. Taken together, these findings support the ITQ-CG as an effective tool for identifying PTSD in youth, with the caveat that using this measure as currently designed may result in patterns of DSM-5 vs. ICD-11 findings different than previously expected based on prior literature. This further underscores the need for additional investigations into the best diagnostic models for PTSD among diverse populations exposed to an array of potentially traumatic events.

## Data Availability

The data that support the findings of this study are available from the corresponding author (B.D.) on reasonable request.

## Abbreviations

PTSD: Posttraumatic stress disorder
DSM-5: Diagnostic and Statistical Manual of Mental Disorders, Fifth Edition
ICD-11: International Classification of Disease, Eleventh Edition
ITQ-CG: International Trauma Questionnaire Child and Adolescent Caregiver Report Version
RI-5: UCLA PTSD Reaction Index for DSM-5, Parent/Caregiver Version
